# Childhood Pneumonia in Low- and Middle-Income Countries: A Systematic Review of Prevalence, Risk Factors, and Healthcare-Seeking Behaviors

**DOI:** 10.7759/cureus.57636

**Published:** 2024-04-04

**Authors:** Selvi M, Sasi Vaithilingan

**Affiliations:** 1 Community Health Nursing Department, Vinayaka Mission's Research Foundation, Salem, IND; 2 Community Health Nursing Department, Vinayaka Mission's College of Nursing, Puducherry, IND

**Keywords:** healthcare-seeking behavior, community-based interventions, risk factors, epidemiology, community-acquired pneumonia, pediatric pneumonia

## Abstract

Childhood pneumonia is a major contributor to illness and death in children under the age of five globally. Despite advancements in medical science, the burden of pediatric community-acquired pneumonia (CAP) remains high, particularly in low- and middle-income countries. This systematic review aims to synthesize existing literature on the prevalence, risk factors, and healthcare-seeking behaviors associated with pediatric CAP to inform the development of targeted community-based interventions.

An extensive search of various databases such as Medline, EMBASE, Web of Science, Cochrane, PubMed, PubMed Central, Helinet, SpringerLink, Google Scholar, and Biomed Central was performed, resulting in 65 potentially relevant studies. After a thorough evaluation process, 25 studies were selected for the final analysis. These selected studies offered valuable information on the epidemiology, risk factors, and healthcare-seeking behaviors associated with childhood pneumonia.

The review revealed that environmental factors such as indoor air pollution, overcrowding, and exposure to tobacco smoke are significant risk factors for pediatric pneumonia. Additionally, socioeconomic factors, including poverty and a lack of access to clean water and sanitation, contribute to the vulnerability of children to this disease. Poor healthcare-seeking behaviors, driven by limited knowledge and awareness of pneumonia symptoms and treatment, further exacerbate the situation.

The review also highlighted the critical role of vaccination, particularly against *Haemophilus influenzae* type b (Hib) and pneumococcus, in preventing pneumonia. However, gaps in vaccination coverage and challenges in accessing healthcare services remain barriers to effective pneumonia control.

In light of these findings, the review recommends the implementation of community-based interventions that address the multifaceted determinants of pediatric pneumonia. These interventions should focus on improving environmental conditions, enhancing access to preventive measures such as vaccination, and promoting better healthcare-seeking behaviors through education and awareness campaigns. It is essential for healthcare providers, policymakers, and community members to collaborate in developing and implementing culturally appropriate and sustainable interventions. This cooperation aims to lessen the impact of pneumonia on children and their families.

## Introduction and background

The solidification and discharge of fluid in the lung tissue characterizes pneumonia, a serious respiratory illness also known as a lower respiratory tract infection. Epidemiological studies have shown that, beyond bacterial and viral agents, various factors contribute to the incidence and severity of pneumonia, with environmental determinants playing a pivotal role [1,2]. These environmental factors include air pollution, exposure to tobacco smoke, and living conditions that predispose individuals to respiratory infections [1-3].

Community-acquired pneumonia (CAP) is a term used to describe this condition when it occurs outside of hospitals or other healthcare settings. CAP is marked by acute lung damage and the crucial involvement of alveoli, the tiny air sacs vital for gas exchange. This leads to sepsis and damage to these alveoli, resulting in reduced oxygen intake. In children, the predominant clinical symptoms of CAP include rapid breathing and chest indrawing, alongside cough, fever, weight loss, fatigue, dyspnea, and occasionally, diarrhea or vomiting [4,5]. The leading causative agents of CAP in children are identified as respiratory syncytial virus (RSV), *Haemophilus influenzae*, and *Streptococcus* *pneumoniae*. These pathogens position pneumonia as one of the foremost causes of morbidity and mortality among children under five years of age. The World Health Organization (WHO, 2019) and UNICEF (2021) highlight pneumonia and diarrhea as the principal causes of infant mortality, with pneumonia accounting for 14% of all pediatric deaths globally [5,6].

The study emphasizes the multifaceted risk factors elevating pneumonia prevalence, categorized into certain, probable, and possible factors. These include birth weight, nutrition, and breastfeeding practices, which are crucial in the early life stages. Environmental factors like indoor pollution, especially from using biomass fuels for cooking and heating, and the status of vaccinations are also important. Social determinants like living conditions, parental health behaviors, and access to healthcare services are important in influencing the likelihood and seriousness of pneumonia in children [7,8]. The interplay of these factors, alongside coexisting conditions and nutritional deficiencies, underscores the critical need for primary prevention strategies tailored specifically to children [9-11].

New studies have found that *Mycobacterium pneumoniae* and RSV are the main pathogens that cause pneumonia [10-12]. Key symptoms of pneumonia include low oxygen levels and more work being done to breathe. Amoxicillin-clavulanate stands out as an effective treatment for CAP, with studies showing a positive link between CAP management and maternal awareness of respiratory infections, literacy, and health-seeking behaviors. These behaviors are pivotal in addressing risk factors and are instrumental in lowering disease and death rates in children under the age of five [12].

This current paper seeks to investigate the risk factors for pediatric pneumonia in the community, with a focus on understanding the prevalence and causes of CAP in children. The goals of this study are to find out how common CAP is among children and pinpoint the risk factors linked to it. By identifying these factors, we can create and apply effective measures to lessen the impact of pneumonia on children's health.

## Review

Methodology

In this study, a systematic review was employed as the research methodology, which involved several key stages to ensure a thorough and evidence-based analysis of the literature on pneumonia in children. Initially, the research questions were formulated to guide the review process, focusing on specific aspects such as prevalence, risk factors, and interventions. To collect pertinent studies, various databases were used, including PubMed, EMBASE, Cochrane, Web of Science, Medline, Google Scholar, SpringerLink, Biomed Central, Helinet, and PubMed Central. The selection of studies was based on predefined inclusion and exclusion criteria to ensure relevance and quality. The findings from the selected studies were then compiled and organized systematically, summarizing key data and conclusions. Finally, the conclusions were interpreted based on the strength and consistency of the evidence across the studies. A total of 65 studies were collected from various sources, with a limited number of qualitative research studies included to enhance the sensitivity of the review. The systematic review approach ensured a comprehensive, unbiased, and evidence-based examination of the existing literature on pneumonia in children.

**Figure 1:**
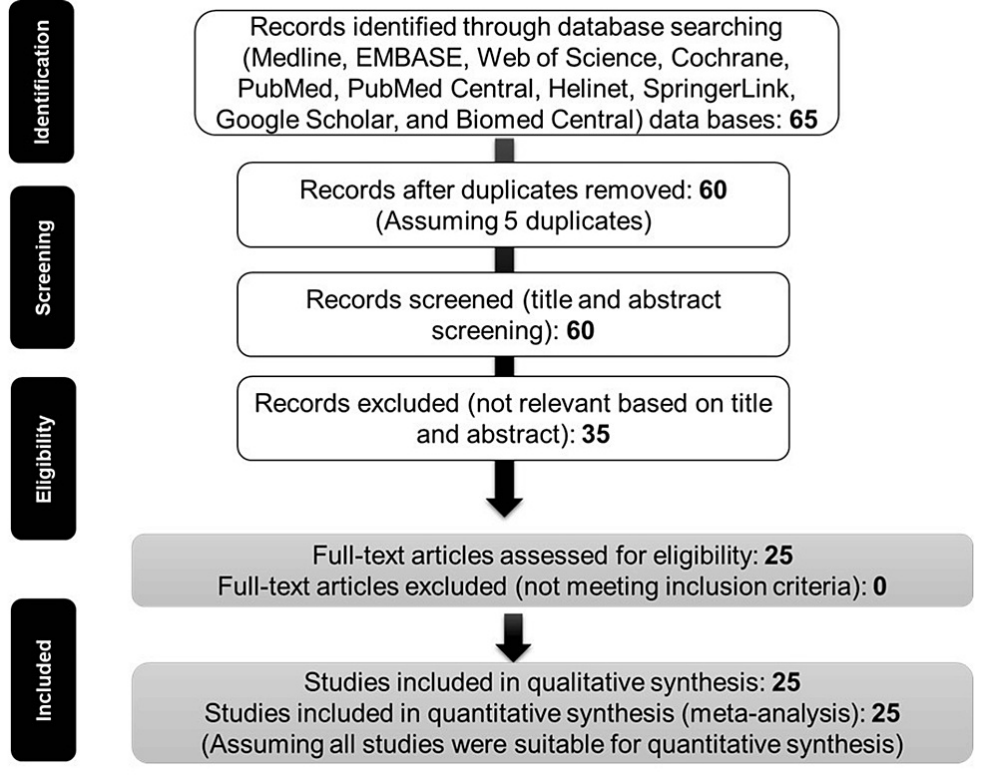
The PRISMA flow diagram shows the steps taken to pick studies for the review, starting with finding 65 possible studies and ending with choosing 25 studies for detailed examination. PRISMA: Preferred reporting items for systematic reviews and meta-analyses

The selection of studies for inclusion in this systematic review was based on a set of predefined criteria. The inclusion criteria encompassed studies related to pneumonia, specifically primary research studies that contribute to the main body of knowledge. The research needed to focus on children aged one to five years and could be of either qualitative or quantitative nature. Only studies with findings published in English and conducted globally were considered. Additionally, the research had to be carried out in both hospital and home care settings. On the other hand, the exclusion criteria ruled out studies with poorly disclosed methodologies and conclusions, research works released prior to 2013, and reviews of previously published works (Figure 1). This approach ensured a comprehensive and relevant selection of studies for the review.

Summary of the findings

A total of 25 studies addressing pneumonia in children met the selection criteria, comprising three qualitative and 22 quantitative studies. Data were extracted and compiled into tables, covering various research types including case-control, prospective, retrospective, cross-sectional, and community-based surveys. The studies were conducted in diverse global settings including India, Canada, Ethiopia, Lebanon, Pakistan, South Africa, Sudan, Tanzania, and the United States.

Analysis of pediatric pneumonia prevalence

Data on incidence, risk factors, and health-seeking behavior were extracted and synthesized from the combined results of these 25 studies. Both qualitative and quantitative research were assessed. A retrospective study involving 369 children revealed that the proadrenomedullin peptide could be used to predict the severity of pneumonia, with levels detected at 0.70 nmol/L (0.55-1.04) in severe cases [13]. A Taiwanese cross-sectional study identified RSV as the most common pathogen in children under two, while *S. pneumoniae* (31.6%) was prevalent in severe cases among children aged two to five [14]. In Lucknow, the incidence of CAP was reported to be 86.50 cases per 1000 persons. Another study in Ujjain, India, found that severe pneumonia affected 64% of the 270 children diagnosed with CAP, with incidence linked to various factors such as premature birth, incomplete vaccination history, and inadequate living conditions [15]. A community survey in South Korea showed seasonal and annual patterns in severe pneumonia incidence, influenced by RSV and *Mycoplasma pneumoniae* in children older and younger than two years, respectively [16]. A retrospective analysis by Daniel et al. (2019) highlighted a high January death rate in children aged one month to 14 years [17]. A four-month cross-sectional survey reported that 24.58% (824 out of 3351) of children aged two to 59 months received care in the preceding year, with 4% hospitalized [18]. A cross-sectional study in Shandong discovered that the proadrenomedullin peptide worsened the condition of CAP [19]. Several studies identified risk factors affecting pneumonia prevalence.

Analysis of studies on risk factors

Regarding risk factors, the main concerns for the prevalence and severity of CAP are identified risk factors. Data from various studies on factors affecting children's CAP incidence were analyzed. A cross-sectional study of 94 children in Pakistan using conventional culture-identified atypical bacteria, viruses, and Mycobacterium pneumonia as etiological agents of pediatric pneumonia [20]. Risk factors for children's pneumonia in Ethiopia included older mothers, housewives, lack of a separate kitchen, recent acute lower respiratory tract infections, and parental asthma [21]. In Mysore, Karnataka, children's pneumonia was associated with proximity to busy highways, vehicle dealerships, gas stations, ground floor parking, living on lower floors, and inadequate ventilation [22]. A study in Tanzania identified modifiable risk factors for CAP in children, including underweight, failure to exclusively breastfeed for six months, and dirty cooking fuel [23]. The study by Al-Dalfi et al. (2023) aimed to understand which social and demographic factors are related to how severe pneumonia is in children under five years old in Wasit Governorate. They looked at 477 children in five hospitals from October 2022 to May 2023. Most of the children (81%) had pneumonia, with 14% having very severe cases and 5% having severe cases. The study found that children most at risk of getting pneumonia were usually between one and 11 months old boys and living in cities. Most of their parents had finished primary school, and almost all mothers were married and housewives. Many fathers worked for themselves, and about a quarter of the families lived in crowded homes. Also, 65% of the families did not have much money. The study showed that how severe a child's pneumonia is can be related to what their father does for work and how much money the family has [24]. A mass cross-sectional survey in India found that 0.49% of children under five reported having pneumonia monthly, linked to unclean fuel use, partial immunization, and mothers' lack of knowledge about hygiene and child care [25]. A case-control study suggested that male gender may be a protective factor against childhood pneumonia, while household density and vaccination status increased the risk [26]. To reduce children's mortality and morbidity, studies on pneumonia's risk factors and etiological characteristics advised emphasizing parents' or caregivers' health-seeking actions.

Analysis of health-seeking behavior data

Information from studies on CAP incidence and risk factors indicated the importance of prioritizing caregivers' or parents' health-seeking behaviors. The collective analysis of healthcare-seeking data revealed various findings. A qualitative study found that mothers and other caregivers often did not seek medical attention when symptoms worsened, relied on traditional medicine as a first line of treatment, were unaware of transmission risks, and only sought help when the condition worsened [27]. A survey examining the knowledge, attitudes, and practices of mothers with children suffering from pneumonia revealed that 74.3% had insufficient knowledge, 91% thought pneumonia could be treated at home, 50.7% gave over-the-counter medications, and 34% used home remedies and herbs [28]. The study by File et al. (2004) tested whether a higher dose of amoxicillin-clavulanate (2000 with 125 mg) was as effective as the standard dose (875 with 125 mg) in treating adult pneumonia when administered twice daily for seven days. The research involved 633 pneumonia patients and determined that the most frequent bacteria responsible for the infection were *S. pneumoniae*, *Staphylococcus aureus*, and *H. influenzae* (Table 1).

**Table 1:** Analysis of health-seeking behavior data for CAP in children. CAP: community-acquired pneumonia

Name of the study	Key findings	Reference number
Purwati et al., 2021	Mothers and caregivers often relied on traditional medicine, were unaware of transmission risks, and sought medical help only when the condition worsened.	[27]
Saeed & Awadalla, 2020	74.3% of mothers had insufficient knowledge about pneumonia, 91% believed it could be treated at home, 50.7% used over-the-counter medications, and 34% used home remedies and herbs.	[28]
File et al., 2004	Tested the efficacy of a higher dose of amoxicillin-clavulanate compared to the standard dose for adult pneumonia treatment.	[29]
Kifle et al., 2023	Identified handwashing, exclusive breastfeeding, zinc supplementation, recent diarrhea, and upper respiratory tract infections as significant factors associated with pneumonia in children.	[30]
Kajungu et al., 2023	Caregivers had some knowledge about pneumonia, but there was room for improvement. Education, marital status, and income were linked to better knowledge.	[31]
Simieneh et al., 2019	Only 48.8% of mothers sought healthcare for their sick children, with several factors influencing their decision.	[32]
Temsesgen et al., 2023	62.2% of caregivers delayed seeking medical attention for pneumonia, with several factors associated with the delay.	[33]

They checked how well the treatments worked by seeing if patients got better (clinical success) and if the germs were gone (bacteriological success). For the higher dose, 90.3% of patients got better, and 86.6% had no more germs. For the standard dose, 87.6% of patients got better, and 78.4% had no more germs. The study found that the higher dose of amoxicillin-clavulanate is just as good at treating pneumonia as the standard dose, without causing more side effects (Table 1) [29].

The research conducted by Kifle et al. (2023) aimed to focus on the determinants of pneumonia in children under five years of age at Hiwot Fana Specialized Hospital in Eastern Ethiopia. It was carried out from October 1 to November 30, 2022; this hospital-based case-control study encompassed 348 children, comprising 116 cases and 232 controls. Data were gathered using a pre-tested questionnaire and analyzed using the Statistical Package for the Social Sciences (SPSS) software (version 25) (IBM Corp., Armonk, NY). The study found that several factors were significantly associated with pneumonia, including handwashing before feeding a child, breastfeeding exclusively for the first six months, zinc supplementation, recent diarrhea, and upper respiratory tract infections. These findings suggest that pneumonia in young children could be prevented or managed with minimal cost by focusing on appropriate health education and interventions targeting these risk factors [30]. Efforts to mitigate illness and death caused by pneumonia in children under five years old are largely reliant on the actions of caregivers (Table 1).

The study by Kajungu et al. (2023) aimed to understand how much caregivers in Eastern Uganda know about pneumonia, what they think about it, and how they act to prevent or treat it. The investigator's group talked to 649 caregivers of kids under five years old. Authors found that while most caregivers knew pneumonia is a disease that affects children, they did not know much about how it spreads, its signs and symptoms, its risk factors, or how to treat it. Approximately 28% possessed good knowledge, 36% had moderate knowledge, and 35% displayed poor knowledge. More than half (57%) had a positive attitude, and most (74.1%) said they had good practices to prevent or treat pneumonia (Table 1).

Caregivers who were older, had more education, and were married tended to know more about pneumonia. Having a positive attitude was linked to being over 35 years old and having a stable source of income. Good practices were more common among female caregivers, Muslims, and farmers. The study suggests that caregivers of young children have some knowledge about pneumonia, but there is room for improvement. Better education, being married, and having a steady income were linked to better knowledge, while being female, Muslim, and a farmer were linked to better practices. The researchers recommend targeted efforts to give caregivers, especially those with lower incomes and less education, the skills and knowledge they need to identify and manage pneumonia in children, considering cultural and religious beliefs [31]. The study conducted in Northwest Ethiopia aimed to explore the healthcare-seeking behavior of mothers and caregivers for their children. It surveyed 410 mothers in the Aneded district and discovered that only 48.8% of them sought healthcare when their child was sick, with just 27% doing so within a day of noticing the illness. Several factors influenced the likelihood of seeking healthcare: awareness of childhood illnesses significantly increased the likelihood, understanding the importance of early treatment also had a notable impact, and having children under 24 months slightly increased it. Conversely, not perceiving the illness as severe decreased the likelihood of seeking healthcare (AOR=0.17, 95% CI: 0.09-0.30). The study concluded that the overall level of healthcare-seeking behavior was low among mothers in this region and suggested focusing on education about the signs of illness and the importance of early treatment, especially for those with young children, to improve this behavior [32]. The research conducted by Temsesgen et al. (2023) aimed to explore the reasons behind the delay in seeking healthcare for pneumonia in children under five years old by caregivers in Nekemte town, Ethiopia. Conducted from March 1st to April 5th, 2022, the study involved 410 caregivers of children aged two to 59 months. It was found that 62.2% of caregivers delayed seeking medical attention for pneumonia. The delay was associated with several factors, including residing in rural areas, having a child aged 12 months or older, earning a monthly income of less than 1000 Ethiopian birr, not having health insurance, resorting to self-medication, lacking adequate knowledge about pneumonia, perceiving the illness as mild, and having no prior hospital admissions for the child. The study emphasizes the need to improve caregivers' awareness and provide comprehensive health education to encourage timely healthcare-seeking behavior and promote the use of health insurance (Table 1) [33].

These studies highlight the critical role of caregivers' and parents' health-seeking behaviors in managing CAP in children. Factors such as knowledge, beliefs, financial constraints, and access to healthcare facilities significantly impact timely and appropriate care-seeking actions [34-41].

Discussion

The prevalence of pediatric pneumonia and the associated health-seeking behaviors in developing countries have been the focus of numerous studies due to their significant impact on child morbidity and mortality. In this discussion, we will look into the results of three recent studies that help us understand these topics in different areas and also review 25 studies that focus on pneumonia in children. The first study, conducted in East Africa, aimed to estimate the pooled prevalence of pneumonia among children under five years of age and identify its associated factors through a systematic review and meta-analysis [42]. The study found that the prevalence of pneumonia in this region was alarmingly high, with a considerable variation across different countries. Factors such as malnutrition, lack of exclusive breastfeeding, indoor air pollution, and low socioeconomic status were identified as significant contributors to the high prevalence of pneumonia. These findings highlight the need for targeted interventions to address the underlying risk factors and reduce the burden of pneumonia in East African countries.

In India, a study explored the prevalence, potential determinants, and treatment-seeking behavior of acute respiratory infections (ARI) among children under five years of age, using data from the National Family Health Survey, 2019-2021 [43]. The study revealed a substantial prevalence of ARI in this age group, with significant variations across different states. Factors such as the age of the child, nutritional status, household air pollution, and access to healthcare services were found to be associated with the occurrence of ARI. Moreover, the study highlighted that a considerable proportion of affected children did not seek formal healthcare services, indicating a gap in health-seeking behavior that needs to be addressed.

A study in rural Tanzania explored the prevalence of childhood illnesses, including pneumonia, and the health-seeking behavior patterns among caregivers. It revealed that pneumonia was quite common, with caregivers frequently turning to informal healthcare providers or self-medicating instead of visiting formal health facilities. The study highlighted that factors like the proximity to health facilities, the economic status of households, and caregiver traits impacted health-seeking behavior [44]. This research emphasizes the need to enhance access to healthcare services and increase caregiver awareness about the significance of timely and proper care for childhood illnesses. In addition to these studies, a comprehensive analysis of 25 studies addressing pneumonia in children revealed various insights into the prevalence, risk factors, and health-seeking behaviors associated with pediatric pneumonia. The studies, conducted in diverse global settings including India, Canada, Ethiopia, Lebanon, Pakistan, South Africa, Sudan, Tanzania, and the United States, covered various research types including case-control, prospective, retrospective, cross-sectional, and community-based surveys. Risk factors for children's pneumonia were found to be multifaceted, including environmental factors, socioeconomic status, and access to healthcare services. The health-seeking behavior of caregivers was identified as a critical factor in the management of pneumonia, with studies highlighting the need for improved awareness and access to healthcare facilities.

Recommendations

Based on the comprehensive review of literature on pneumonia in children, it is recommended to implement targeted interventions addressing the underlying risk factors such as malnutrition, lack of exclusive breastfeeding, indoor air pollution, and low socioeconomic status. Efforts should be made to increase awareness among caregivers and parents about the signs, symptoms, and risk factors of pneumonia, with a focus on promoting timely and appropriate health-seeking behaviors. Improving access to healthcare services is crucial, including ensuring the availability of essential medicines, vaccines, and healthcare facilities, as well as promoting the use of health insurance to reduce financial barriers. Continued research and surveillance are needed to monitor the trends and determinants of pneumonia in children, which will help in identifying emerging risk factors and guiding policy decisions. Collaboration between governments, non-governmental organizations, healthcare providers, and communities is essential for the successful implementation of interventions. It is also important to consider cultural and contextual factors in designing and implementing strategies. Alongside treatment, there should be a strong emphasis on preventive measures such as vaccination, improving indoor air quality, and promoting good hygiene practices.

To further investigate the multifactorial nature of pediatric pneumonia, exploring a wider range of potential risk factors, including environmental, socioeconomic, and genetic factors, is recommended. Expanding research to include a broader range of geographic settings, particularly in regions with high pneumonia burdens, can ensure findings are globally representative. Incorporating qualitative research methods can provide deeper insights into health-seeking behaviors, caregiver perceptions, and barriers to accessing healthcare. Designing and evaluating intervention studies, such as vaccination programs, public health campaigns, and environmental improvements, can test the effectiveness of strategies aimed at reducing the incidence and severity of pediatric pneumonia. Enhancing the quality of data collection and reporting, including the use of standardized diagnostic criteria and robust statistical methods, is essential. Investigating and addressing barriers to healthcare access, including financial constraints, distance to facilities, and cultural beliefs, can improve timely and appropriate care-seeking behavior. Translating research findings into policy and practice is crucial, with the development of evidence-based guidelines and strategies for the prevention, diagnosis, and management of pediatric pneumonia. Engaging communities in the research process and the implementation of interventions can increase awareness, promote preventive measures, and encourage appropriate health-seeking behavior. By addressing these recommendations, the morbidity and mortality associated with pneumonia in children can be significantly reduced, particularly in regions where the burden of this disease is highest.

## Conclusions

The studies reviewed in this discussion underscore the substantial burden of pediatric pneumonia in developing countries, characterized by a high prevalence of the disease and its associated risk factors. These studies highlight the necessity for targeted interventions aimed at addressing the underlying determinants of pneumonia, such as malnutrition, indoor air pollution, and insufficient exclusive breastfeeding. These interventions should encompass nutritional programs to combat malnutrition, environmental health measures to mitigate indoor air pollution, vaccination campaigns to protect against key pathogens, and health education initiatives to enhance caregivers' knowledge and awareness. By implementing these targeted interventions, we can address the fundamental causes of pediatric pneumonia, improve health-seeking behaviors among caregivers, and significantly reduce the morbidity and mortality associated with the disease, ultimately leading to better health outcomes for children in developing countries.

## References

[1] Debnath SK, Debnath M, Srivastava R (2022). Opportunistic etiological agents causing lung infections: emerging need to transform lung-targeted delivery. Heliyon.

[2] Debnath SK, Srivastava R, Debnath M, Omri A (2021). Status of inhalable antimicrobial agents for lung infection: progress and prospects. Expert Rev Respir Med.

[3] Ding L, Wang J, Cai S, Smyth H, Cui Z (2021). Pulmonary biofilm-based chronic infections and inhaled treatment strategies. Int J Pharm.

[4] Mackenzie G (2016). The definition and classification of pneumonia. Pneumonia (Nathan).

[5] Scott JA, Wonodi C, Moïsi JC (2012). The definition of pneumonia, the assessment of severity, and clinical standardization in the Pneumonia Etiology Research for Child Health study. Clin Infect Dis.

[6] Quach A, Spence H, Nguyen C, Graham SM, von Mollendorf C, Mulholland K, Russell FM (2022). Slow progress towards pneumonia control for children in low-and-middle income countries as measured by pneumonia indicators: a systematic review of the literature. J Glob Health.

[7] Dida GO, Lutta PO, Abuom PO, Mestrovic T, Anyona DN (2022). Factors predisposing women and children to indoor air pollution in rural villages, Western Kenya. Arch Public Health.

[8] Odo DB, Yang IA, Dey S (2022). Ambient air pollution and acute respiratory infection in children aged under 5 years living in 35 developing countries. Environ Int.

[9] Vardoulakis S, Giagloglou E, Steinle S (2020). Indoor exposure to selected air pollutants in the home environment: a systematic review. Int J Environ Res Public Health.

[10] Rudan I, O'Brien KL, Nair H (2013). Epidemiology and etiology of childhood pneumonia in 2010: estimates of incidence, severe morbidity, mortality, underlying risk factors and causative pathogens for 192 countries. J Glob Health.

[11] le Roux DM, Zar HJ (2017). Community-acquired pneumonia in children - a changing spectrum of disease. Pediatr Radiol.

[12] Nascimento-Carvalho CM (2020). Community-acquired pneumonia among children: the latest evidence for an updated management. J Pediatr (Rio J).

[13] Florin TA, Ambroggio L, Brokamp C (2021). Proadrenomedullin predicts severe disease in children with suspected community-acquired pneumonia. Clin Infect Dis.

[14] Chi H, Huang YC, Liu CC (2020). Characteristics and etiology of hospitalized pediatric community-acquired pneumonia in Taiwan. J Formos Med Assoc.

[15] Kasundriya SK, Dhaneria M, Mathur A, Pathak A (2020). Incidence and risk factors for severe pneumonia in children hospitalized with pneumonia in Ujjain, India. Int J Environ Res Public Health.

[16] Lee E, Kim CH, Lee YJ (2020). Annual and seasonal patterns in etiologies of pediatric community-acquired pneumonia due to respiratory viruses and Mycoplasma pneumoniae requiring hospitalization in South Korea. BMC Infect Dis.

[17] Farrar DS, Awasthi S, Fadel SA (2019). Seasonal variation and etiologic inferences of childhood pneumonia and diarrhea mortality in India. Elife.

[18] Awasthi S, Pandey CM, Verma T, Mishra N (2019). Incidence of community acquired pneumonia in children aged 2-59 months of age in Uttar Pradesh and Bihar, India, in 2016: an indirect estimation. PLoS One.

[19] Chang J, Liu W, Huang C (2018). Residential ambient traffic in relation to childhood pneumonia among urban children in Shandong, China: a cross-sectional study. Int J Environ Res Public Health.

[20] Shakeel S, Iffat W, Qamar A (2021). Pediatricians’ compliance to the clinical management guidelines for community-acquired pneumonia in infants and young children in Pakistan. Healthcare (Basel).

[21] Bazie GW, Seid N, Admassu B (2020). Determinants of community acquired pneumonia among 2 to 59 months of age children in Northeast Ethiopia: a case-control study. Pneumonia (Nathan).

[22] Kumar KJ, Ashok Chowdary KV, Usha HC, Kulkarni M, Manjunath VG (2018). Etiology of community acquired pneumonia among children in India with special reference to atypical pathogens. Lung India.

[23] Ngocho JS, de Jonge MI, Minja L, Olomi GA, Mahande MJ, Msuya SE, Mmbaga BT (2019). Modifiable risk factors for community-acquired pneumonia in children under 5 years of age in resource-poor settings: a case-control study. Trop Med Int Health.

[24] Al-Dalfi MH, Al Ibraheem SA, Al-Rubaye AK (2023). The severity of pneumonia and its association with socio-demographic factors among children under five years old in Wasit governorate hospitals, Iraq. J Public Health Afr.

[25] Gothankar J, Doke P, Dhumale G (2018). Reported incidence and risk factors of childhood pneumonia in India: a community-based cross-sectional study. BMC Public Health.

[26] Fonseca Lima EJ, Mello MJ, Albuquerque MF, Lopes MI, Serra GH, Lima DE, Correia JB (2016). Risk factors for community-acquired pneumonia in children under five years of age in the post-pneumococcal conjugate vaccine era in Brazil: a case control study. BMC Pediatr.

[27] Purwati NH, Rustina Y, Supriyatno B (2021). Knowledge and healthcare-seeking behavior of family caregivers of children with pneumonia: a qualitative study in an urban community in Indonesia. Belitung Nurs J.

[28] Saeed EIM, Awadalla A (2020). Knowledge, attitude and, practice among mothers of under-five children about acute lower respiratory tract infections in a locality in Khartoum urban area, Sudan. J Environ Sci Public Health.

[29] File TM Jr, Lode H, Kurz H, Kozak R, Xie H, Berkowitz E (2004). Double-blind, randomized study of the efficacy and safety of oral pharmacokinetically enhanced amoxicillin-clavulanate (2,000/125 milligrams) versus those of amoxicillin-clavulanate (875/125 milligrams), both given twice daily for 7 days, in treatment of bacterial community-acquired pneumonia in adults. Antimicrob Agents Chemother.

[30] Kifle M, Yadeta TA, Debella A, Mussa I (2023). Determinants of pneumonia among under-five children at Hiwot Fana specialized hospital, Eastern Ethiopia: unmatched case-control study. BMC Pulm Med.

[31] Kajungu D, Nabukeera B, Muhoozi M (2023). Factors associated with caretakers' knowledge, attitude, and practices in the management of pneumonia for children aged five years and below in rural Uganda. BMC Health Serv Res.

[32] Simieneh MM, Mengistu MY, Gelagay AA, Gebeyehu MT (2019). Mothers' health care seeking behavior and associated factors for common childhood illnesses, Northwest Ethiopia: community based cross-sectional study. BMC Health Serv Res.

[33] Temsesgen D, Wordofa B, Tesfaye T, Etafa W (2023). Delay in seeking healthcare for pneumonia and associated factors among mothers/caregivers of children aged 2-59 months in public health facilities in Nekemte town, Ethiopia. BMC Pediatr.

[34] Tannous R, Haddad RN, Torbey PH (2020). Management of community-acquired pneumonia in pediatrics: adherence to clinical guidelines. Front Pediatr.

[35] Wetzke M, Kopp MV, Seidenberg J (2019). PedCAPNETZ - prospective observational study on community acquired pneumonia in children and adolescents. BMC Pulm Med.

[36] Awasthi S, Kumar D, Mishra N, Agarwal M, Pandey CM (2019). Effectiveness of various communication strategies for improving childhood pneumonia case management: a community based behavioral open labeled trial in rural Lucknow, Uttar Pradesh, India. BMC Public Health.

[37] Kerai S, Nisar I, Muhammad I (2019). A community-based survey on health-care utilization for pneumonia in children in peri-urban slums of Karachi, Pakistan. Am J Trop Med Hyg.

[38] Alexandra G, Mihai C (2018). Study on parental perception and approach regarding community acquired pneumonia in preschool children from Romania. Ro J Pediatr.

[39] Awasthi S, Verma T, Agarwal M (2017). Developing effective health communication messages for community-acquired pneumonia in children under five years of age: a rural North Indian qualitative study. Clin Epidemiol Glob Health.

[40] Parikh K, Biondi E, Nazif J, Wasif F, Williams DJ, Nichols E, Ralston S (2017). A multicenter collaborative to improve care of community acquired pneumonia in hospitalized children. Pediatrics.

[41] Bham SQ, Saeed F, Shah MA (2016). Knowledge, attitude and practice of mothers on acute respiratory infection in children under five years. Pak J Med Sci.

[42] Beletew B, Bimerew M, Mengesha A, Wudu M, Azmeraw M (2020). Prevalence of pneumonia and its associated factors among under-five children in East Africa: a systematic review and meta-analysis. BMC Pediatr.

[43] Varghese JS, Muhammad T (2023). Prevalence, potential determinants, and treatment-seeking behavior of acute respiratory infection among children under age five in India: findings from the National Family Health Survey, 2019-21. BMC Pulm Med.

[44] Kanté AM, Gutierrez HR, Larsen AM (2015). Childhood illness prevalence and health seeking behavior patterns in rural Tanzania. BMC Public Health.

